# Scleroderma and type 1 diabetes: a rare association

**DOI:** 10.11604/pamj.2018.30.77.15720

**Published:** 2018-05-29

**Authors:** Nassiba Elouarradi, Nawal El Ansari

**Affiliations:** 1 Service of Endocrinology, Diabetology and Metabolic Diseases, University Hospital of Marrakech, Marrakech, Morocco

**Keywords:** Scleroderma, diabetes, morhea

## Image in medicine

The association of type 1 diabetes and systemic scleroderma is rarely reported in the literature, the pathogenesis of this association is unknown, interferon seems to have a major role in being an immunomodulator and inhibitor of collagen production, and it is also involved in autoimmune pathology. Note that this association could be at the origin of a difficulty of passage of the insulin in the sites of injections, responsible for a major glycemic imbalance. We report the case of a 26-year-old patient, who had been diabetic for 6 years on insulin, who was referred for a glycemic imbalance, who had a clinical examination objectifying multiple morphea lesions on the roots of the thighs, arms, abdomen and thorax. A cutaneous biopsy was performed, showing a sclerodermiform appearance with significant fibrosis without sign of malignancy, result rather in favor of a scleroderma.

**Figure 1:**
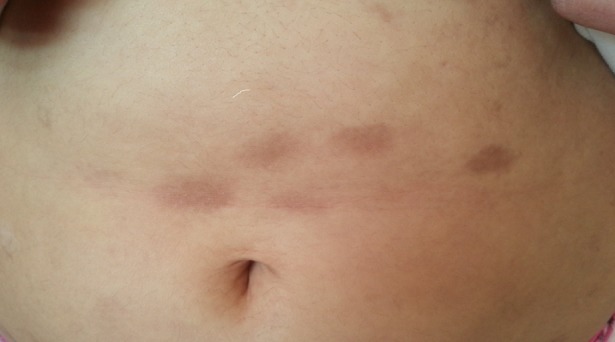
Localized morhea in the abdomen

